# Point of care ultrasound-detected postoperative diaphragmatic dysfunction and its association with pulmonary complications after thoracic surgery: protocol for a prospective, observational study

**DOI:** 10.3389/fmed.2026.1759942

**Published:** 2026-03-02

**Authors:** Jiajun Li, Chunlin Tang, Yanqing Huang, Xi Zheng

**Affiliations:** Department of Anesthesiology, Guangzhou Medical University Cancer Hospital, Guangzhou, China

**Keywords:** diaphragm, diaphragmatic dysfunction, diaphragmatic ultrasound, perioperative period, postoperative pulmonary complications, thoracic surgery, ultrasound application, ultrasound indicators

## Abstract

**Introduction:**

Postoperative pulmonary complications (PPCs) significantly impact recovery after thoracic surgery. Postoperative diaphragmatic dysfunction (PDD) may be a key risk factor but remains under-recognized, particularly regarding comparative data between video-assisted thoracoscopic surgery (VATS) and robot-assisted thoracic surgery (RATS). This study aims to investigate the association between PDD detected by point-of-care ultrasound (POCUS) and PPCs following minimally invasive thoracic surgery.

**Methods:**

This is a single-center, prospective observational study conducted at the Affiliated Cancer Hospital of Guangzhou Medical University. A total of 148 patients scheduled for elective pulmonary resection (VATS or RATS) will be enrolled. Diaphragmatic function will be assessed using POCUS at four time points: 1 day preoperatively (T0), 30 min post-extubation (T1), and on postoperative day 1 (T2) and 3 (T3). The primary measurements include diaphragmatic excursion (DE) and diaphragmatic thickening fraction (DTF). The primary endpoint is the incidence of PDD (defined as DE < 10 mm or negative) on postoperative day 1. Secondary endpoints include the incidence and severity of PPCs within 7 days postoperatively (defined by European Perioperative Clinical Outcome criteria), serial changes in DE and DTF, pain scores, and quality of recovery (QoR-15). Propensity score matching and multivariate regression will be used to adjust for confounders.

**Conclusion:**

This study will elucidate the relationship between POCUS-detected PDD and PPCs in patients undergoing VATS and RATS. The findings may support the integration of diaphragmatic ultrasound into perioperative risk stratification and guide targeted preventive strategies to improve postoperative outcomes.

**Trial registration:**

https://www.chictr.org.cn, ChiCTR2500103734.

## 1 Introduction

Lung resection is an important treatment for treating benign and malignant lung diseases. However, postoperative pulmonary complications (PPCs) remain a major factor affecting patient recovery. It has been reported that the incidence of PPCs in thoracic surgery including thoracotomy and minimally invasive surgery is as high as 20–45% (1–5) and this incidence range is summarized based on studies with complete postoperative 7-day follow-up and PPCs defined in accordance with the European Perioperative Clinical Outcome (EPCO) criteria (6). PPCs not only prolong hospital stays but also increase medical costs and mortality rates (7, 8). In recent years, minimally invasive surgical methods, such as video-assisted thoracoscopic surgery (VATS) and robot-assisted thoracic surgery (RATS) have been widely adopted due to their advantages of less trauma and faster recovery. As compared to the standard thoracotomic approach, minimally invasive surgeries decrease surgical complications, postoperative pain, and the overall incidence of PPCs.

However, postoperative diaphragmatic dysfunction (PDD), as a potential risk factor for PPCs, has not been fully recognized in lung resection. The term diaphragmatic dysfunction includes eventration, weakness and diaphragmatic paralysis (9). The diaphragm is the primary inspiratory muscle, and its function directly affects postoperative respiratory mechanics and expectoration ability. Studies have shown that the incidence of PDD after thoracic surgery can be as high as 12.5–68%, and it is closely related to PPCs such as pulmonary infection and atelectasis (7, 10, 11). Traditional diagnosis includes radiography, fluoroscopy, pulmonary function tests, and stimulation of the phrenic nerve, with some limitations including exposure to ionizing radiation, intense patient cooperation and less sensitivity or specificity. However, point-of-care ultrasound (POCUS) offers a noninvasive and dynamic approach to assess perioperative diaphragmatic function. Ultrasound has shown to be useful for the detection of diaphragmatic dysfunction, with a high sensitivity (93%) and specificity (100%) for diaphragmatic neuromuscular disease, and it has been suggested as the technique of choice for assessing diaphragmatic movement on suspicion of malfunctioning (9). Spadaro et al. first introduced POCUS in thoracic surgery. They defined DD as diaphragmatic excursion (DE) < 1 cm and demonstrated an increased risk of PPCs in patients with early PDD (OR = 5.5) (7). Recent evidence further highlights a lower postoperative diaphragmatic excursion difference (DED) after video-assisted thoracoscopic surgery (VATS) can serve as an additional marker, which helps predict the risk of PPCs (11). Additionally, Daniel et al. reported that PDD predominantly occurs on the surgical side, persists until postoperative 3rd day, and independently prolongs hospital stays (OR = 1.3) (12). Diaphragmatic thickness (DT) and thickening fraction (DTF) are also critical metrics; DT reduction >10% or DTF <20% are defined as thresholds for clinically relevant atrophy in critically ill patients according to an expert consensus in 2022 (13), with the point that preoperative diaphragmatic atrophy (DT < 0.2 cm) significantly increases PPCs risk.

Some studies suggest that the outcomes of RATS are similar to or even better than those of VATS surgery (5, 14). Geraci et al. reported that the incidence of early postoperative complications in 253 patients who underwent RATS lung resection was only 6.4% (15). However, this study lacked long-term follow-up and the definition of PPCs may differ from that in many previous studies, which may have resulted in an underestimation of the incidence of PPCs. RATS, with its three-dimensional high-definition vision, flexible instruments and tremor filtering function, theoretically can reduce the intraoperative traction of the phrenic nerve and chest wall trauma (16); however, RATS-specific intraoperative factors such as CO2 insufflation (capnothorax) induce an artificial increase in intrathoracic pressure, which exerts direct mechanical and physiological effects on the diaphragm: the elevated intrathoracic pressure antagonizes the abdominal-to-thoracic pressure gradient generated by diaphragmatic contraction, directly reducing the effective excursion amplitude of the diaphragm; sustained intrathoracic hypertension compresses the intrathoracic microvasculature, impairs local microcirculatory perfusion of the diaphragmatic muscle fibers, and hinders oxygen supply and metabolite excretion of the muscle tissue; in addition, the diaphragm needs to maintain a higher contractile tension to overcome the increased intrathoracic pressure during the surgical period, which easily induces early fatigue of the diaphragmatic muscle fibers. These changes may alter diaphragmatic excursion and contractility in the perioperative period, in contrast to VATS, which does not require artificial CO2 insufflation to establish a pneumothorax and maintains intrathoracic pressure at a physiological level without the above-mentioned diaphragm function interference factors—an aspect rarely addressed in previous studies. Patients can be discharged on the first day after RATS lung resection, and both the clinical effect and patient satisfaction are excellent (15). Early research by Mahieu et al. showed that the proportion of emergency conversion to thoracotomy during RATS was significantly lower than that of traditional video-assisted thoracoscopic surgery (VATS) (3.6% vs. 14.3%), suggesting its potential safety advantage (17). Despite these advances, current studies predominantly focus on single-timepoint assessments of diaphragmatic function or single minimally invasive surgical modality, lacking longitudinal data comparing VATS and RATS across the perioperative period.

Based on these, we conducted a prospective cohort design, combined with perioperative multi-time point POCUS monitoring of diaphragm function, to explore the correlation between PDD and PPCs after minimally invasive thoracic surgery (VATS and RATS). Additionally, the risk factors for PPCs will be screened out, by integrating perioperative diaphragm ultrasound monitoring with ARISCAT, we also aim to verify whether this predictive efficacy will be improved.

## 2 Methods and analysis

### 2.1 Study design

**Figure 1:**
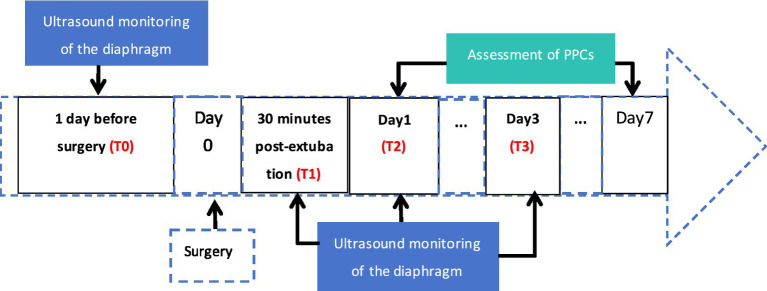
Timeline of the research process.

**Figure 2:**
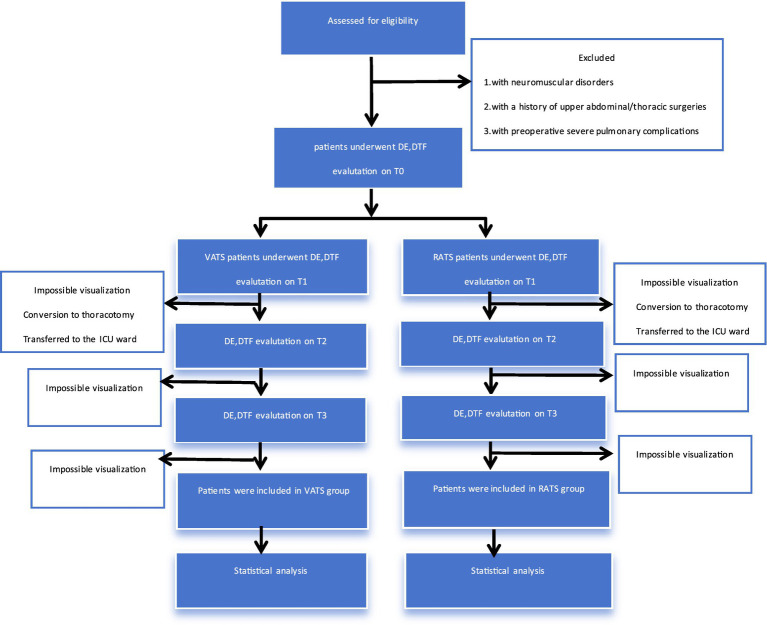
Flow chart of the study.

This was a non-randomized, single-center, prospective observational study conducted at the Affiliated Cancer Hospital of Guangzhou Medical University. One hundred and forty-eight patients who underwent elective pulmonary resection for lung disease between July 2025 and May 2026 were included. Figure 1 is the study timeline. Bilateral diaphragm function was monitored by ultrasound at 4 time periods, 1st day before surgery (T0), 30 min post-extubation in the Post-anesthesia Care Unit (PACU) (T1), 1st day after surgery (T2), and 3rd day after surgery (T3). This standardized T1 measurement window will be uniformly applied to all enrolled patients in the study. Diaphragm thickness at the end of inhalation (DTei), diaphragm thickness at the end of exhalation (DTee), and DE during voluntary calming respiration were recorded at each time period. The occurrence of patients’ PPCs was tracked and observed during the 7 day postoperative period, and related clinical variables were recorded, respectively. Figure 2 is the trial flow chart.

### 2.2 Inclusion and exclusion criteria

Inclusion criteria:

(1) Age between 18 and 80 years.
(2) American Society of Anesthesiologists (ASA) Physical Status I–III.
(3) BMI between 18.5 and 30 kg/m^2^.
(4) Scheduled to undertake RATS/VATS lobectomy/segmentectomy.
(5) Signed the informed consent form.

Exclusion criteria:

(1) Those with neuromuscular disorders (e.g. Guillain-Barré syndrome, myasthenia gravis).
(2) Those with a history of upper abdominal/thoracic surgeries.
(3) Those with preoperative severe pulmonary complications (such as pulmonary infection, pleural effusion, atelectasis, respiratory failure, or cardiopulmonary edema).
(4) Those of which we cannot obtain a satisfactory ultrasound imaging or do not have space for placement of the ultrasonic probe.
(5) Those with diaphragm insufficiency preoperatively (determined by a combination of preoperative pulmonary function tests, point-of-care ultrasound (POCUS) assessment, and clinical manifestations such as persistent dyspnea or decreased exercise tolerance, without relying on phrenic nerve stimulation).
(6) When minimally invasive surgical approaches convert to thoracotomy in operation.
(7) Those with severe cardiac diseases, or hepatic and renal dysfunction (in such patients, drug metabolism is significantly impaired, and residual muscle relaxants and anesthetics may persist after intubation, thereby inhibiting diaphragmatic movement).
(8) Withdrawal from the study or withdrawal of informed consent during the study.
(9) Immediately transferred to the ICU ward after surgery due to major adverse events such as intraoperative massive hemorrhage and refractory hypoxemia requiring mechanical ventilation (such patients will be transferred to the ICU with endotracheal intubation for continued respiratory support, resulting in significantly prolonged mechanical ventilation time, which will further impair diaphragmatic function).

### 2.3 Diaphragmatic ultrasound protocol

#### 2.3.1 Operating process

1st day before surgery (T0), 30 min post-extubation in the Post-anesthesia Care Unit (PACU) (T1), 1st day after surgery (T2), and 3rd day after surgery (T3), diaphragmatic ultrasound was performed with the patient in a semi-recumbent position in bed with the head of the bed elevated by 30° performing quiet breathing. We recorded DE during voluntary calming respiration (7) (excluding deep inspiration and sniff manoeuver, as these two maneuvers are associated with poor compliance in postoperative patients due to pain and chest drain tube interference), we evaluated both left and right hemidiaphragms. To standardize the acoustic window across time points, preoperatively (T0) we mark the probe position on the chest wall using skin-friendly, non-irritating markers, recording the distance from the midclavicular line/anterior axillary line and rib interspace. Postoperatively, if surgical dressings, chest tubes, or subcutaneous emphysema block the original site, we adjust the probe to the nearest adjacent interspace while maintaining the same anatomical orientation relative to the diaphragm dome, documenting the adjustment in the case report form. All diaphragmatic ultrasound measurements at T0–T3 time points are performed by the same fixed team consisting of 1 experienced independent anesthesiologist and 1 resident trainee, both of whom have completed 3 months of specialized training in diaphragmatic ultrasound, to minimize inter-observer variability. The independent anesthesiologist always performed the measurements by first marking the best acoustic window on the patient’s chest for the trainee’s observation of the measurements.

**Figure 3:**
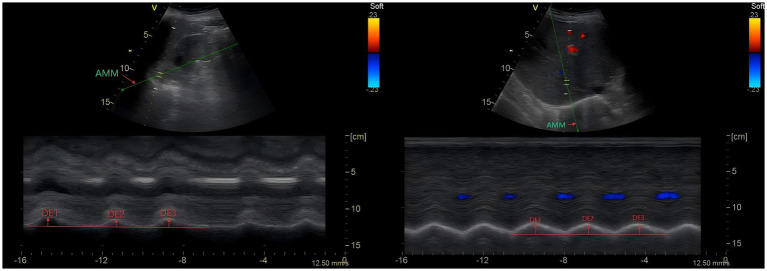
Diaphragm ultrasonography1: DE of left and right hemidiaphragm.

DE was assessed using a 3.5–5 Mhz convex ultrasound probe with the liver and spleen as the ultrasound windows for the right and left hemidiaphragm, respectively. To show the right hemidiaphragm, the probe was placed between the midclavicular and anterior axillary line intercostals, whereas for the left hemidiaphragm, a subcostal or low intercostal probe position was chosen between the anterior midaxillary line. For left hemidiaphragm measurement, if the splenic acoustic window is insufficient (unclear diaphragm visualization for ≥3 consecutive breathing cycles), we adjust the probe angle, increase the imaging depth (up to 6 cm), or use the left subcostal approach with the patient slightly rotated to the right. If visualization remains unsuccessful, we document the left hemidiaphragm as “unmeasurable” and focus on the right hemidiaphragm (especially the surgical side, if unilateral resection) for analysis. The hemidiaphragm probe line was selected using the two-dimensional mode (B mode). The probe was fixed to the chest wall with the ultrasound probe located medially, cephalad, and dorsally so that the ultrasound beam reached the posterior portion of the hemidiaphragm dome at an angle as close to 90 as possible, and then the ultrasonography machine was switched to the motion mode (M mode). Using anatomical M-mode (AMM), during inspiration, the normal diaphragm contracted and moved caudally toward the transducer; this was recorded as an upward movement tracked by the motion mode and was regarded as a diaphragmatic excursion during inspiration, which was measured on the vertical axis from baseline to maximum inspiratory height on the frozen image, and the resultant was the DE (Figure 3) (18, 19). Similarly, the left hemi-transverse diaphragm mobility was measured under the splenic window.

**Figure 4:**
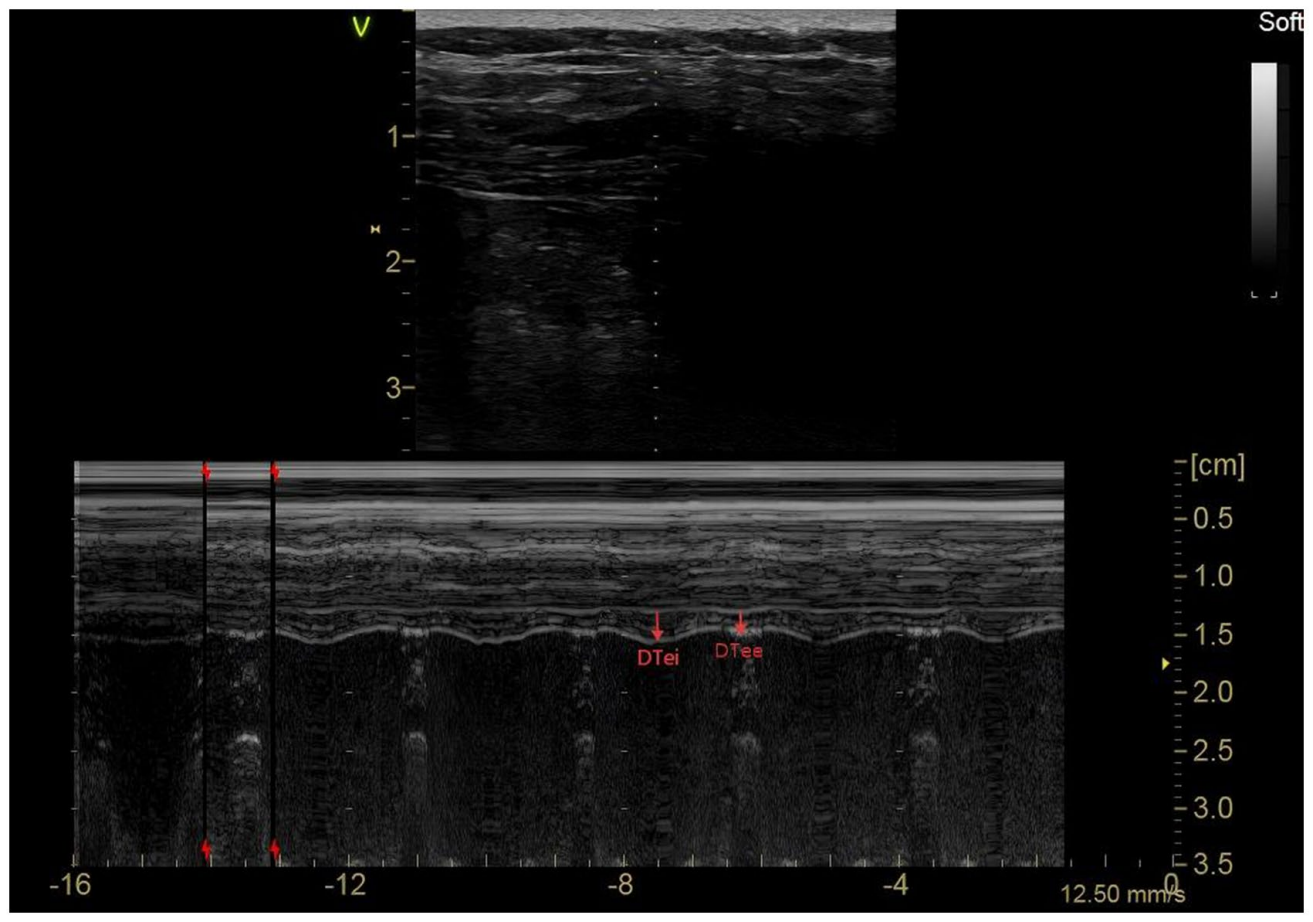
Diaphragm ultrasonography 2: DTei and DTee.

In the anterior axillary/mid-axillary line along the rib margins, in B-mode, at a depth of 2–4 cm from the skin, a two-dimensional sagittal image (Figure 4) was obtained, in which a hypoechoic diaphragm located between two layers of hyperechoic lines (pleura and peritoneum) could be seen, and the thoracic and peritoneal spacing perpendicular to the direction of the fibers was measured as the DT (20–22). With the patient’s respiratory movements, the DTei and the DTee can be measured separately. DTR (diaphragm thickening ratio) = DTei-DTee; DTF = (DTei-DTee)/DTee × 100%.

#### 2.3.2 Diagnosis to PDD

DE values less than 10 mm or negative (7) [based on our preliminary experiments and others’ studies, the strong association between this critical value and diaphragmatic function has been validated (23)] is our primary measurement. Diaphragmatic weakness is determined where there is decreased DE with or without paradoxical movement during the sniff manoeuver. DTF values below 20% were interpreted as indicative of diaphragm paralysis (9, 13, 24).

### 2.4 Anesthesia and perioperative management

#### 2.4.1 Anesthesia induction

All patients are transferred to the operation room without any premedication and receive standard monitoring, including electrocardiogram (ECG), noninvasive and invasive blood pressure, peripheral oxyhemoglobin saturation (SpO_2_), respiratory rate (RR), bispectral index (BIS), surgical pleth index (SPI), and partial pressure of end-tidal carbon dioxide (PETCO_2_) intraoperatively. Central venous catheterization is performed in the right internal jugular vein before anesthesia induction. A forced-air warming blanket and core temperature monitoring are routinely used to maintain the patient’s temperature between 36 and 37 °C. Preoxygenation for 5 min, then anesthesia was induced using propofol 1.5–2.0 mg/kg, midazolam 0.08 mg/kg, sufentanil 0.4 μg/kg, and rocuronium 0.9 mg/kg. After artificially assisting respiration for 3 min, all patients were intubated with a Shiley™ double lumen tube to achieve lung isolation and fibreoptic bronchoscopy was used to confirm correct positioning, and then the patient was placed in a supine position or a lateral position, and the fiberoptic bronchoscope was used to check the correct position of the tracheal catheter again.

#### 2.4.2 Maintenance of anesthesia

Since there is some influence of anesthesia mode or drugs on diaphragm function, it is important to homogenize the anesthesia management (25). Using PCV-VG mode, two-lung mechanical ventilation sets tidal volume at 6 ~ 8 mL/kg (ideal body weight), with 50%FiO2 to maintain SpO2 ≥ 95% (routine 100% FiO2 avoided to reduce absorption atelectasis risk), and the 50%FiO2 is the minimum feasible concentration to maintain the target SpO2 in this ventilation mode. For one-lung mechanical ventilation, a protective lung ventilation strategy is applied: tidal volume set to 4 ~ 5 mL/kg, respiratory rate adjusted appropriately, PEEP at 3 cmH2O, FiO2 is titrated according to the principle of using the lowest feasible concentration to maintain SpO2 ≥ 90%, and FiO2 titrated to maintain SpO2 ≥ 90% (up to 100% only for persistent hypoxemia), and driving pressure limited to ≤15 cmH2O to ensure PETCO2 ranges 35–45 mmHg. Capnothorax pressure during RATS is maintained at ≤8 mmHg to minimize diaphragmatic compression effects, and after one-lung ventilation completion and before resuming two-lung ventilation, the standardized lung recruitment maneuver technique of sustained inflation is performed for all patients in both VATS and RATS cohorts (sustained inflation at 20–25 cmH2O for 10 s) to reduce atelectasis. Anesthesia was maintained with remifentanil (0.15–0.3 μg/kg/min), propofol (1–4 mg/kg/h) and dexmedetomidine (0.5 μg/kg/h), combined with inhalation of sevoflurane 1–2%, with the anesthetics titrated to maintain a BIS index between 40 and 60 (7, 26) and avoid hypotension or hypertension [defined as a greater than 20% decrease or increase in mean arterial pressure from baseline (27)]. Intermittent intravenous injections of rocuronium are used to maintain muscle relaxation. All patients are managed according to a standardized protocol of enhanced recovery after thoracic surgery.

#### 2.4.3 Analgesia and postoperative pain assessment

##### 2.4.3.1 Multimodal analgesia regimen

Before the end of surgery, 50 mg flurbiprofen axetil is administered intravenously. At the end of surgery, the surgeon administers intercostal nerve block (site: T3–T7, 0.2% ropivacaine with epinephrine 1:200000, 3–5 mL at each site, using repeat doses) (28); Then a patient-controlled intravenous analgesia (PCIA) pump will be installed. PCIA pump setting: background infusion condition is hydromorphone 0.002 mg/kg/h, the bolus setting is maintained at 0.002 mg/kg, the lockout interval is 10 min (29, 30). When NRS > 4, oxycodone 10-20 mg was administered intravenously to remedy analgesia, twice per day; the standard dose was 20 mg, which was reduced to 10 mg if the patient developed nausea (31). Once the patient’s pain is relieved, perform a diaphragm ultrasound examination on the same day only when NRS ≤ 4. Ondansetron is used to prevent postoperative nausea and vomiting (PONV). Postoperative pain was assessed by the same two physicians throughout the study period using an 11-point numerical rating scale (NRS 0 = no pain; 10 = worst possible pain) immediately after extubation (T1), 1st day after surgery (T2) and 3rd day after surgery (T3), respectively. Additionally, Ramsay sedation scores were performed by the same two physicians at the same time (we incorporated this score into the outcome measurement to clarify whether there is heterogeneity in postoperative sedation levels among the target population and to explore whether or how postoperative sedation levels influence diaphragmatic function or pulmonary outcomes).

#### 2.4.4 Postoperative extubation

Patients are transferred to the PACU with endotracheal intubation, and 4 mg/kg of sugammadex is intravenously pushed within 10s upon PACU arrival. Extubation will be performed when all the following criteria are met: (1) the patient was fully awake and could perform simple commands; (2) the pharyngeal reflex, swallowing reflex, and coughing reflex were fully restored; (3) the patient’s voluntary respiration was regular, and the tidal volume was 6–7 mL/kg (ideal body weight); (4) SpO_2_ was greater than 96% on FiO_2_ of 0.4 or less and PETCO_2_ was less than 45 mmHg; (5) hemodynamic stability; (6) core temperature was 36.5 °C or higher; (7) no evidence of early surgical complications; (8) adductor hallucis muscle train-of-four ratio (TOFr, T4/T1) ≥ 0.9 (extubation criteria follow international expert consensus or guidelines. However, diaphragm may recover faster than peripheral muscles; a TOFr ≥ 0.9 at adductor hallucis does not fully guarantee complete diaphragmatic recovery. This is precisely the reason why we set up T1–30 min post-extubation for POCUS measurements to ensure as complete a recovery of diaphragmatic function as possible).

#### 2.4.5 Quality of postoperative recovery

Quality of Recovery Scale-15 (QoR-15) scores were performed by specially trained nurse anesthetists on preoperatively (T0), 1st day postoperatively (T2) and 3rd day postoperatively (T3).

#### 2.4.6 Safety assessment

Detailed documentation of adverse events during the perioperative process: intraoperative hypotension (SBP < 90 mmHg or less than 20% of basal value) and sinus bradycardia (HR < 50 bpm), hypertension (SBP > 180 mmHg or more than 20% of basal value) and tachycardia (HR > 100 bpm), and delayed awakening (90 min after discontinuation of drugs). Other rare anesthesia-related complications include myocardial infarction, unstable angina, new onset arrhythmias, pulmonary edema, heart failure, respiratory failure, pneumonia, stroke, venous thrombosis, pulmonary embolism, renal failure, hepatic failure, gastrointestinal hemorrhage, and more.

#### 2.4.7 Assessment of PPCs

Postoperatively, pulmonary complications from postoperative 1st day to 7th day were recorded by a supervising physician who was unaware of the diaphragmatic ultrasound parameters. According to European Perioperative Clinical Outcome (EPCO) definitions (6), PPCs were defined as respiratory-related adverse events occurring in the postoperative period, which mainly included: respiratory tract infections, respiratory failure, pleural effusion, pulmonary atelectasis, and pneumothorax, bronchospasm, aspiration pneumonia (Table 1). In addition, PPCs were categorized as mild, moderate, or severe using pragmatic severity grading (Table 2).

**Table 1:** Postoperative pulmonary complications (PPCs).

Complications	Definition
Respiratory tract infection	The patient is receiving antibiotics for a suspected respiratory infection that meets one or more of the following criteria: new or altered sputum, new or altered cloudy lungs, fever, and a white blood cell count of >12 × 109/L
Respiratory failure	Postoperative room air inhalation PaO2 < 8 kPa (60 mmHg), PaO2: FI02 ratio < 40 kPa (300 mmHg), or arterial oxygen saturation < 90% measured by pulse oximetry, requiring oxygen therapy
Pleural effusion	Chest radiographs show blunting of the rib-diaphragm angle, loss of clear contour of the ipsilateral hemidiaphragm in the upright position, evidence of displacement of adjacent anatomical structures, or (in the supine position) blurred shadows of one hemithorax with preserved vascular shadows
Atelectasis	Lung turbidity, displacement of the mediastinum, hilar or hemidiaphragm toward the involved area, compensatory hyperinflation of the adjacent non-atelectatic lungs
Pneumothorax	Air in the pleural cavity, no vascular bed around the visceral pleura
Bronchospasm	Newly recognized expiratory wheeze treated with bronchodilators
Aspiration pneumonia	Acute lung injury following inhalation of regurgitated contents

**Table 2:** Pragmatic severity grading criteria (severity grading).

Severity	Definition	Clinical processing needs
Mildly	Causes only temporary damage and usually requires no special treatment	May require observation or basic support only (e.g., oxygen)
Moderately	More serious complications, but usually not leading to permanent damage or functional limitations	Clinical intervention required (e.g., antibiotics, noninvasive ventilation)
Severe	Significantly prolonged hospitalization, resulting in permanent functional limitations or death	Requires aggressive treatment (e.g., mechanical ventilation, ICU admission)

Mild: transient postoperative hypoxemia requiring short-term oxygenation.

Moderate: pneumonia requiring intravenous antibiotic therapy that has not progressed to respiratory failure.

Severe: acute respiratory distress syndrome (ARDS) requiring tracheal intubation and ICU monitoring.

### 2.5 Allocation, evaluation and blindness

The choice of the surgical modality was mainly determined by the surgeon based on technical and oncological reasons. The team of surgeons who performed the surgery had performed more than 50 cases of RATS previously. It has been reported that, after surgeons had completed about 20 cases of robotic-assisted lobectomy, the operative time had been reduced significantly, and the rate of complications had stabilized, which can be considered as basic mastery of the technique, accumulating 50–90 cases of experience to achieve technical proficiency (14).

Diaphragmatic ultrasound was performed twice by 2 observers (1 independent anesthesiologist and 1 resident trainee, who were not involved in intraoperative anesthesia management) to quantify inter-observer variability, both of whom had undergone 3 months of specialized training in diaphragmatic ultrasound and were familiar with ultrasound theory and practice, using smart ultrasound instrumentation. PPCs are recorded only if two supervising physicians (who were unaware of the diaphragmatic ultrasound parameters) diagnose the same PPCs in the same patient.

### 2.6 Objectives

#### 2.6.1 Primary

To describe the incidence of postoperative diaphragmatic dysfunction (PDD) and explore its association with postoperative pulmonary complications (PPCs) after minimally invasive thoracic surgery (VATS and RATS).

#### 2.6.2 Secondary

To identify potential risk factors (such as surgical technique) of PPCs and to evaluate the clinical application of diaphragm ultrasound examination.

### 2.7 Endpoints

#### 2.7.1 Primary endpoint

Incidence of PDD (DE values less than 10 mm or negative) at 1 day postoperatively.

#### 2.7.2 Secondary endpoints

(1) Incidence and severity of PPCs at 7 days postoperatively; (2) T0-T3 diaphragm function at different time points (DTF, DE and the difference of DE between T1/2/3 and T0). (3) NRS scores and Ramsay sedation scores at different time points. (4) QoR-15 scores at different time points.

### 2.8 Sample size calculation

Sample size is recalculated based on the primary predictive objective (logistic regression for the association between PDD and PPCs). Based on the literature review of perioperative PPCs incidence in minimally invasive thoracic surgery (VATS/RATS) with standardized EPCO complication definitions and complete 7-day postoperative follow-up and our pilot study, the baseline PPC incidence of 37% was selected as the core assumption for this power analysis and sample size calculation; Assuming a PDD incidence of 30% postoperatively (7, 12), a PPCs incidence of 37% in the study population (1–5, 15, 32), an odds ratio (OR) of 3.0 for PDD predicting PPCs (7, 28), alpha = 0.05, power = 0.80, and 10% dropout rate, a minimum of 148 patients in total is required. Sample size estimation is performed using PASS software (PASS 15 Power Analysis and Sample Size Software (2017). NCSS, LLC. Kaysville, Utah, United States)^1^ with logistic regression parameters accounting for covariates (age, BMI, ASA classification), and the above parameter selection fully aligns with the literature review results of PPCs incidence in minimally invasive thoracic surgery, ensuring the consistency between clinical evidence and statistical methodology.

### 2.9 Statistical analysis

Percentages will be calculated for dichotomous data and analyzed by the Pearson chi-square test or Fisher exact test. Continuous variables will be expressed as mean standard deviation (Mean ± SD) and median (ranging from 25th to 75th percentiles), and Student’s *t*-test was used for comparison between groups; The paired sample *t*-test is used for comparison of the same index. Propensity Score Matching (PSM) will be performed to balance baseline characteristics (e.g., age, BMI, ASA classification, tumor complexity) between the VATS and RATS groups (1:1 matching ratio, caliper width 0.2 SD) to mitigate selection bias. A baseline characteristics table will be added as Table 1 in the final manuscript, which will present the demographic, clinical and surgical-related baseline data of the VATS and RATS groups before and after PSM matching, so as to clearly show the balance effect of the matching process on the baseline characteristics between the two groups. Multivariate regression models will further adjust for residual confounding variables not fully balanced by PSM. Spearman and Pearson correlation analyses are used for the correlation analysis of normal distribution variables and non-normal distribution variables, respectively. Differences in diaphragmatic function parameters (DE, DTF) across multiple time points (T0–T3) will be analyzed using Linear Mixed-Effects Models (LMM) to account for within-subject correlation of repeated measures and robustly handle missing data. Differences in DEs at different times in the same subject were assessed by the Wilcoxon signed-rank test for matched data. Associations between postoperative DE reduction and clinically significant perioperative variables were analyzed using binary logistic regression analysis and reported as estimated crude advantage ratios and relative 95% CI. Similarly, binary logistic regression with stepwise variable selection (forward method) will be used to explore risk factors for PPCs, following the “Rule of Ten” (at least 10 events per candidate variable) to avoid model overfitting. To determine the predictive power of ultrasound parameters for PPCs, ROC curve analysis will be performed to validate the predefined PDD threshold (DE < 10 mm) rather than seeking a new cut-off, evaluating its discriminatory ability for PPCs. For the correlation analysis between diaphragmatic ultrasound parameters (DE, DTF) and ARISCAT scores, Pearson correlation analysis will be adopted if both variables conform to normal distribution, and Spearman correlation analysis will be used if the distribution is non-normal. On this basis, a combined predictive model for PPCs will be constructed by integrating ultrasound parameters (DE, DTF) and ARISCAT scores, and Net Reclassification Index (NRI) and Integrated Discrimination Improvement (IDI) will be used to quantify the improvement of the combined model in the discriminatory ability of PPCs prediction compared with the ARISCAT score single-factor model; the area under the ROC curve (AUC) of the single-factor model and the combined model will be further compared to verify the predictive efficacy of the integrated approach.

For missing data including unmeasurable left hemidiaphragm ultrasound measurements, Multiple Imputation (MI) will be used to handle incomplete observations at any time point (T0–T3), with 20 imputed datasets generated. Listwise deletion will not be adopted to avoid selection bias and preserve statistical power. All statistical analyses were performed using SPSS software Version 26.0 (IBM, United States) and R software (Version 4.3.0) with packages “lme4” (for LMM) and “mice” (for MI).

### 2.10 Data collection and processing

We also recorded other parameters: age, gender, BMI, ASA classification, NYHA classification, metabolic equivalents (MET), preoperative comorbidities (COPD, chronic heart disease, chronic liver disease, metabolic disorders), pulmonary function, smoking history, Hb, SpO2, albumin, presence of respiratory infections in the previous month, ARISCAT score (33, 34), Arozullah score, surgical approach (VATS/RATS), surgical site and side, duration of surgery, extent of surgery, duration of intraoperative one-lung ventilation, amount of intraoperative rehydration and bleeding, extent of lymph node dissection, duration of postoperative chest tube drainage (to explore its potential association with diaphragmatic function), DE values during voluntary calming respiration at each time point (T0–T3), acoustic window adjustment records (if any) and left/right hemidiaphragm measurability status, duration of postoperative hospitalization and adverse events during the perioperative process. All data will be recorded in the same Excel sheet. Ultrasound images and videos will be copied from the ultrasound machine and stored on a removable hard disk for viewing.

## 3 Conclusion

This study will elucidate the relationship between POCUS-detected PDD and PPCs in patients undergoing VATS and RATS. The findings may support the integration of diaphragmatic ultrasound into perioperative risk stratification and guide targeted preventive strategies to improve postoperative outcomes.

## 4 Discussion

The higher incidence of PPCs in thoracic surgery patients is significantly associated with PDD (7, 9, 11, 35). We will use POCUS to specifically investigate the correlation between these two factors in minimally invasive surgery patients. As surgical techniques have evolved, the opportunities for clinical applications of RATS in thoracic surgery are increasing. In fact, according to a review of the Society for Thoracic Surgery General Thoracic Surgery Database 2023 (STS-GTSD), more than 80% of lobectomies, segmental resections, and wedge resections are performed through minimally invasive approaches (5). The feasibility of RATS lobectomy has been confirmed by several relevant studies in the last 5 years; compared to open thoracotomy, RATS seems to offer the same advantages as the VATS access (16). We will further evaluate the effects of these two surgical approaches on diaphragm function in surgical patients.

We emphasize the importance of performing diaphragm ultrasound in perioperative patients with thoracic surgery, a high-risk group for PPCs, to demonstrate the clinical efficacy of POCUS during the perioperative period. This includes preoperative risk stratification and early postoperative PDD detection. Establishing standardized evaluation criteria is also crucial for guiding the targeted and effective application of POCUS in perioperative care. Our pre-experiments demonstrated that DE has higher feasibility and reproducibility in the postoperative environment when used for PDD exploration. It is reported that interobserver reliability has been shown to yield a bias below 0.1 cm with limits of agreement (LOA) of ±0.3 cm for DE, compared to a bias of −2% and wider LOA of ±21% for DTF (35). Therefore, DE serves as our primary measurement metric for assessing PDD. We acknowledge that DE only recorded during voluntary calming respiration without performing and recording “Deep Inspiration” or “Sniff Maneuver” at each time point is a significant limitation. Observed diaphragmatic dysfunction based on voluntary calming respiration may be exacerbated by postoperative pain and chest tube discomfort, which leads to difficulty in distinguishing between true diaphragmatic weakness and pain-induced restricted breathing (splinting) in the early postoperative period and thus limits the ability to isolate intrinsic diaphragmatic contractility. Additionally, although a peripheral adductor hallucis train-of-four ratio ≥0.9 is the clinical gold standard for safe extubation and sugammadex is administered to reverse neuromuscular blockade, the differential recovery between peripheral muscles and the diaphragm may lead to overestimation of surgical diaphragmatic dysfunction at the T1 (30 min post-extubation) time point; this potential pharmacological noise remains a theoretical possibility that needs to be considered when interpreting early postoperative POCUS results of diaphragmatic function. However, DE is heavily influenced by paradoxical movement and chest wall mechanics after thoracic surgery, which may reflect passive displacement rather than active diaphragmatic dysfunction. In contrast, DTF directly measures diaphragmatic muscle contractility—a key physiological parameter of active function. This limitation is addressed by analyzing DTF as a complementary metric to DE, ensuring comprehensive assessment of diaphragmatic function beyond passive excursion. Furthermore, we will investigate the potential of DTF as a diaphragmatic contraction force to provide additional insights for related research. The ARISCAT score is currently the most widely used tool for assessing the risk of postoperative complications (PPCs) in surgical patients, which showed good discrimination with AUC 0.83 (95% CI 0.79–0.86) on a receiver-operating characteristics curve and the accuracy was also good with a Brier score of 0.19 (34). To demonstrate the incremental predictive value of integrating POCUS parameters with the ARISCAT score, Net Reclassification Index (NRI) and Integrated Discrimination Improvement (IDI) will be used to quantify whether the addition of diaphragmatic ultrasound indicators (e.g., DE, DTF) significantly enhances the discriminatory ability of the ARISCAT score alone. Finally, we hope to further improve the accuracy and applicability of PPCs risk stratification by combining ARISCAT score with perioperative diaphragmatic function detected by POCUS.

However, there are some limitations. For example, this is a single-center study that exclusively involves thoracic surgery; therefore, the generalisability may not be extrapolated. This is a non-randomized clinical study and selection bias may be present, which is mitigated by the inclusion of Propensity Score Matching (PSM) and multivariate covariate adjustment in the statistical analysis plan to balance baseline characteristics between the VATS and RATS groups. Additionally, we decided on a 7-day postoperative follow-up because most patients are discharged around 7 days after surgery. We acknowledge that limiting follow-up to the first 7 postoperative days may underestimate the duration or delayed impact of diaphragmatic dysfunction since postoperative diaphragmatic dysfunction has been reported in the literature to persist beyond the early postoperative period. We stopped POCUS monitoring at T3 (postoperative day 3) based on existing evidence (12, 24, 35) indicating that PDD reaches its peak within the first 72 h post-operatively and is most prominent within the first 3 postoperative days, with the dysfunction index tending to stabilize thereafter; moreover, early PDD (within 3 days) has been identified as a key predictor of subsequent PPCs including those developing at postoperative day 7 (7, 12), even those developing after day 3.

## Footnotes

1
https://ncss.com/software/pass
